# Assessing the efficacy of a culturally adapted cognitive behavioural internet-delivered treatment for depression: protocol for a randomised controlled trial

**DOI:** 10.1186/s12888-018-1634-x

**Published:** 2018-02-27

**Authors:** Alicia Salamanca-Sanabria, Derek Richards, Ladislav Timulak, Leónidas Castro-Camacho, Mónica Mojica-Perilla, Yamilena Parra-Villa

**Affiliations:** 1 E-mental Health Research Group, School of Psychology, Trinity College Dublin, Dublin, Ireland; 2 Clinical Research and Innovation, SilverCloud Health, Dublin, Ireland; 3 Universidad de los Andes, Bogotá, Colombia; 4 Universidad Autónoma de Bucaramanga UNAB, Bucaramanga, Colombia; 5 Aras an Phiarsaigh, School of Psychology, Trinity College Dublin, Dublin, Ireland

**Keywords:** Culturally adapted psychotherapy, Internet-delivered treatment, Depressive symptoms, CBT, College students, Randomised trial

## Abstract

**Background:**

Depression is the principal cause of disability in the world. High prevalence rates of depression in general populations and college students have been found worldwide and in various cultural groups. Low-intensity cognitive behavioural internet-delivered treatment has demonstrated efficacy in high-income-countries (HICs). However little is known of their potential for adaptation and efficacy in low and middle-income countries.

**Methods:**

Study (1) involves the cultural adaptation of the *Space from Depression* cognitive-behaviour internet-delivered programme with an asynchronous support for depressive symptoms. This includes initial researcher/clinician adaptation and the integration of cultural assessment feedback of the programme by a panel of experts and users through the theoretically-based Cultural Relevance Questionnaire (CRQ). Study (2) describes the implementation of the culturally adapted intervention using a randomised controlled trial methodology. The efficacy trial will include an active treatment group and a waiting-list control group of participants meeting eligibility criteria (mild to moderate depression symptoms). The active condition will consist of 7 weekly modules of internet-delivered cognitive behavioural therapy (iCBT) *Space from Depression*, with post-session feedback support. The primary outcome will be the Patient Health Questionnaire (PHQ-9). The study also involves collection of client reported significant events and client satisfaction with the internet-delivered treatment. Data will be collected at baseline and at post-treatment (week 7), and at follow-up (week 20/3 months). Analysis will be conducted on the intention-to-treat basis.

**Discussion:**

The study seeks to establish a theoretically robust methodology for culturally adapting internet-delivered interventions for mental health disorders and to evaluate the efficacy of a culturally adapted internet-delivered treatment for depression in Colombia, with support. The study will be a first contribution to a method for culturally adapting internet-delivered interventions and also a first to examine the efficacy of such an adapted intervention in Latin America.

**Trial registration:**

Clinical trials NCT03062215. Retrospectively registered 14th February 2017.

## Background

High rates of depression have been reported throughout the world [1, 2]. For instance, a 12-month prevalence rate has been estimated in the U.S. at 9.5% and in Europe at 8.5% of the population [3, 4]. In Colombia, 12-month prevalence is between 6.2% and 12.1% [5–8]. The Colombian National Mental Health Survey (2015) has estimated point prevalence of mild to moderate depressive symptoms at 15.6% and severe depressive symptoms at 4.2% of adults. About 50% of the population does not have access to health services [9], while the majority of the population with mental health problems do not have adequate insurance coverage [10].

Despite the necessity to implement psychological interventions in Latin America, there are few studies in this field [11]. More so, treatments are mostly pharmacological, and evidence-based psychological treatments are rarely used. Furthermore, treatments in low and middle income countries (LMICs) are implemented without considering cultural context: very little research has investigated culturally adapted treatments [12], while there is even less research about internet-delivered treatments [13]. Internet delivered psychological treatment may be a suitable alternative to make evidence-based treatments available, especially when nearly 54% of the population use the internet [14].

### Depressive symptoms in college students

Depressive disorders have shown to be more frequent among college students in comparison with the general population [15]. For instance, a systematic review shows a weighted mean prevalence of depression symptoms in college students of 30.6% [16]. These rates are comparable to reports from Latin America [17–20]. In Colombia one study reported high prevalence of depression symptoms (30%) in students [21]. Another recent study showed 36.2% prevalence of depression symptoms in a university sample [22].

Several variables have been associated with vulnerability of college students to depression: changes in lifestyle related to poor sleep habits, eating disorders, economic stressors and family problems [16, 23]. In spite of the high rates of depression within the college population, research studies and treatment for depression in Colombia have received little attention [24]. Recently, a World Mental Health survey reports that only 16.4% of students received treatment for their mental disorder, while in lower-middle/low income countries it was 6.7% [25].

### Treating depression

Psychological treatments for depression have shown better outcomes in comparison with waiting list or placebo and combined treatment is more effective than pharmacotherapy alone [26, 27]. Boumparis, Karyotaki [28] found that psychological treatments can enhance positive affect and decrease negative affect in depression, contributing to better outcomes. A recent meta-analysis showed that Cognitive Behaviour Therapy (CBT) has been extensively researched and it has been demonstrated to be effective for depression [29].

### Low intensity internet-delivered treatment

Low-intensity internet-delivered treatment involves self-guidance, where the content, including CBT techniques that are typically used in face-to-face therapy are delivered using text, pictures, animations, audio files and videos. This type of intervention is highly structured, it involves psychoeducation, activities and supplementary resources, such as a supporter contact via asynchronous message [30].

Internet-delivered interventions have an established empirical base for major depression [31], sub-threshold depression [32], and in maintenance treatments [33–35]. For instance, a systematic review of computer-based interventions for depression found a mean effect size of *d* = 0.78 for the reduction of depressive symptoms post-treatment [34]. A recent review showed how face-to-face psychotherapy and internet-delivered therapy had similar effects [36].

Internet-interventions for depression are available in many languages. Research from Spanish language versions including López-del-Hoyo, Olivan [37] and Montero-Marín, Araya [38] reported positively on the effectiveness of a CBT-based internet-delivered programme for depression. A recent four-year descriptive, naturalistic study monitoring a web-based CBT treatment developed and researched in Mexico indicated that the intervention was useful for depressive symptoms [39]. A feasibility study in Chile found an online treatment to be beneficial, acceptable and feasible [40].

For the most part, internet interventions for depression have been researched in high-income countries. A recent systematic review found that only three articles reported results of RCTs on internet-delivered interventions for mental health conditions in LMICs [13].

### SilverCloud platform evidence: Space from depression

*Space from Depression (Yo puedo sentirme bien – Spanish version)* is a seven module CBT-based intervention for the treatment of depression. Recently, Richards, Timulak [41] reported that *Space from Depression programme* showed lower levels of depression symptoms post-treatment, compared to their baseline score, yielding a large post-treatment effect (*d* = 0.91). They reported significant differences between the treatment group and waiting list control group (*d* = 0.50). The positive outcomes were maintained at three and six months’ follow-up. The treatment was evaluated by users as acceptable and satisfactory [42].

### Culturally adapted psychotherapy (CAP)

Although low-intensity interventions for depression have been developed in western high-income countries and these interventions therefore have been influenced by their specific cultural context (for example, Ireland, UK, Australia), it is important to consider how cultural context may impact on the adaptation for use in LMICs such as Colombia. The authors argue that cultural aspects need to be taken into consideration when translating and adapting interventions to help guarantee similar results to those that have been achieved in high-income countries.

Culturally adapted psychotherapy (CAP) is defined as a systematic change of intervention protocols through consideration of relevance to the culture of the target population and modifies treatment in accordance with clients’ contexts and values [43]. According to Resnicow, Soler [44] cultural sensitivity has two dimensions, top-down adaptations (e.g., language) and bottom-up adaptations (e.g., cultural, social, historical, environmental and psychological aspects), which indicate varying degrees of integration of culture in psychotherapy. Additionally, ecological validity refers to a process that allows generalisation of assumptions derived from research situations to other environments and considers dimensions such as language, persons, metaphors, concepts, contexts, methods, and goals [45]. Further, principles from cross-cultural assessment research (i.e., functional equivalence, conceptual equivalence, linguistic equivalence) contribute to assessing the cultural adaptation of the programme [46].

### Outcomes from CAP research

Meta-analyses have analysed the efficacy of CAP in comparison to non-adapted interventions and other control groups such as waiting list groups in adults. A recent meta-analysis found 136 published studies from previous meta-analysis. The meta-analysis found the overall affect size was *d* = 0.67, which indicates that culturally adapted interventions have better outcomes than other conditions (non-adapted treatment or control group). Likewise treatment effect sizes were significantly larger (*d* = 0.76) in treatment studies than in prevention studies (0.25) [47].

An older meta-analysis by Griner and Smith [48] reported the outcomes from 76 studies of service providers that were classified according to ethnic and/or socio-racial status. The results showed a weighted average effect size of (*d* = 0.45), indicating a moderately strong benefit for the culturally adapted interventions in comparison to control group.

### Culturally adapted internet-delivered treatment

Some studies have considered cultural adaptation for low-intensity internet-delivered treatments. An open trial study [49] examined the preliminary efficacy and acceptability of a culturally modified therapist-guided CBT treatment for anxiety and depression. The results showed that participants improved significantly across all outcome measures, with large within-group effect sizes (Cohen’s *d*) at post-treatment (*d* = 1.08 to 1.74) and 3-month follow-up (*d* = 1.53 to 2.00). Another study [50] showed the efficacy of a cognitive behavioural internet-delivered treatment for depression with weekly telephone support. Treatment group participants reported significantly reduced depressive symptoms (Cohen’s *d* = 0.93) up to 3-months after treatment compared to a control group. Furthermore, Ince, Cuijpers [51] in a randomised control trial demonstrated reductions in depression symptoms in participants using the internet-based, self-help problem-solving intervention, yielding a moderate effect size (Cohen’s *d* = 0.50). Finally, Wagner, Schulz [52] evaluated an internet-based CBT with therapist support for people with posttraumatic stress disorder (PTSD), which showed significant decreases in symptoms of PTSD (*d* = 1.57), depression (*d* = 1.51), and anxiety (*d* = 1.50) and increases in quality of life (*d* = 1.17) post-treatment.

A recent review and meta-analysis on cultural adaptation of minimally guided interventions for common mental disorders, which included e-mental health delivered interventions and bibliotherapy, found that insufficient details on the methodologies used for cultural adaptation were reported. However, these studies showed an increase in effect size of 0.117 (*P* = .04), or a 14% rise in pooled efficacy [53]. Despite the outcomes showing positive post-treatment results, more research is required to develop interventions that incorporate systematic CAP frameworks, especially as the processes for culturally adapting interventions have not been systematic and clearly or fully described in previous research. The current study seeks to examine the acceptability and efficacy of a culturally adapted cognitive behavioural internet-delivered intervention in Colombia - South America.

## Method

### Aims and hypothesis

We aim to assess the efficacy of a culturally adapted cognitive behavioural internet-delivered treatment for college students with depressive symptoms in Colombia. In line with other studies in high-income countries (HICs), and using an already established intervention, we hypothesise that the culturally adapted *Space from Depression* programme will be efficacious, with significant changes within the treatment group and differences post-treatment between the active treatment and the waiting list control group.

#### Overview

The study is a mixed method approach utilising in study 1 quantitative and qualitative methods to assist in the cultural adaptation of the *Space from Depression* intervention and in study 2 a randomised control design to examine the efficacy of the culturally-adapted intervention.

#### Procedure

The programme *Space from Depression* will be culturally adapted for a Colombian population using cultural sensitivity and ecological validity frameworks, including principles from cross-cultural assessment research (Study 1). Once the programme is culturally adapted, it will be tested using a randomised controlled trial methodology (Study 2).

#### Study 1: Cultural adaptation (cultural sensitivity and ecological validity)

The process for *cultural sensitivity* will be developed using a “*top-down”* methodology as described in the framework by Resnicow, Soler [44]. This process will be carried out by the principal researcher (AS) in conjunction with collaborators from SilverCloud Health (the developers), and professional translators and video production teams from Colombia. The process will involve a revision of the translated content in all sections and the inclusion of Colombian expressions for instance, “*guayabo*” (hung over), “*ciclovía*” (bike roads on Sundays) among others. Furthermore, videos and personal stories will be revised to include typical situations and stereotypes for Colombian students, such as Colombian actors and Latin American college stories (e.g. economic problems, pregnancy, relationships).

The process for *ecological validity* will be developed using approaches proposed by Bernal and Sáez-Santiago [45] and Helms [46] and will involve an evaluation of the programme by Colombian users and clinical experts. Through the use of a robust and theoretically-based methodology, the goal is to arrive at an acceptable and culturally adapted intervention that can then be used in a trial to examine its efficacy.

#### Participants

Five student users from a university in Colombia will evaluate the programme. Five experts in clinical psychology from Colombia and two clinical psychology experts from Argentina and Spain, who have experience with internet-delivered interventions and with translation and adaptation of interventions for use in Latin America, will evaluate the programme using the Cultural Relevance Questionnaire (CRQ) that was developed for this study.

#### Eligibility criteria

Students will be selected on the following criteria: a) clinical psychology masters and PhD students at a Colombian University. Experts will be selected on the following criteria: a) native Spanish speaker from Colombia and other Spanish speaking countries; b) PhD in clinical psychology or in psychology; and c) experience in: CBT, depression, research, and low-intensity internet-delivered treatment. These criteria guarantee the rigorousness of the culturally adapted programme.

#### Recruitment and procedure

An email will be sent to masters and PhD psychology students with information about the research study, its aims and a description of the task. Thereafter, they will attend an information session with the researcher (AS) to further explain the study and to collect the informed consent documents from the participants. Participants will then review the programme and complete the CRQ after engaging with all of the treatment modules.

An invitation email will be sent to the seven identified expert researcher/ clinical psychologists in Bogota, Argentina and Spain. They will receive the study information, aims of the research and why it is important, and an invitation to evaluate the *Space from Depression* (*Yo puedo sentirme bien*) programme using the Cultural Relevance Questionnaire. Once they have signed the informed consent document and returned it by email to the research team, they will receive access to the platform and review the programme.

#### Study 2: Efficacy trial

The implementation of the culturally adapted programme and its evaluation among students using a randomised controlled trial methodology at two sites in Colombia.

### Sample size

Power analysis, carried out in G-Power using significance level α = 0.05 and power = 0.80 determined 45 subjects would be required for each test condition (active group and waiting list) to observe a moderate effect (*d* = 0.50) post-treatment between groups [54].

### Eligibility criteria

College students from two universities in Colombia will be included in this study. Both undergraduate and graduate level students from any school at a university in Bogota, and students of psychology, medicine, nursing and education from a university in Bucaramanga will be eligible to participate and will be selected according to the criteria described in Table 1, which mirrors the criteria used in other studies of this type [41], especially initial investigations in new territories.

**Table 1:** Eligibility criteria

Exclusion criteria:
Severe depressive symptoms > 19 on PHQ-9
Suicidal ideation or intent: Score of 2 or above on PHQ-9 question 9 Psychosis
Currently in psychological treatment for depression
On medication for less than 1 month
Alcohol or drugs misuse
Previous diagnosis of an organic mental health disorder
Depression preceding or coinciding a diagnosed medical condition
Inclusion criteria
18-year-old minimum
Mild to moderately severe depressive symptoms: (PHQ-9 score 10–19)

### User recruitment

An email will be sent to college students at two universities. Potential participants will be able to visit a website to receive information about the study, participation criteria, information about the treatment and how to get in contact to proceed with the study.

### Procedure

Once participants have read the study information, informed consent will be obtained from each user before screening and randomisation. Through the SilverCloud platform, participants will be instructed to type their name on the informed consent page, to indicate that they have read and understood the study information and agree to participate in the study. Thereafter, participants will complete measures for screening purposes, including the Patient Health Questionnaire (PHQ-9), Sociodemographic & Clinical History Questionnaire and Generalised Anxiety Disorder-7 (GAD-7). Thereafter, participants will be randomised based on their measures’ score through computer algorithms and they will be assigned to two groups – the active treatment group and waiting-list (WL) control group.

Those assigned to the active treatment will start the internet-delivered treatment immediately for seven weeks, while the WL participants’ treatment will start once the first group has finished the programme. Individuals not meeting the inclusion criteria at baseline assessment will be referred to other appropriate sources of face-to-face support at the student counselling service in the respective universities.

Any interactions or activities completed by the participants on the programme will be considered as a session, and it will be taken in the weekly feedback provided by the supporter. At the beginning of each session the participants will be asked to reflect on their previous session and complete the Helpful Aspects of Treatment Form (HAT). Furthermore, after week 7 the participants will complete Satisfaction with the Treatment (SAT).

Those who do not complete the research measures will receive automatic emails from the platform, to encourage them to use the programme. Likewise, supporters will send an email to the participants who do not complete the programme, and the research team will phone the participants who do not complete the measures.

### Randomisation

**Fig. 1:**
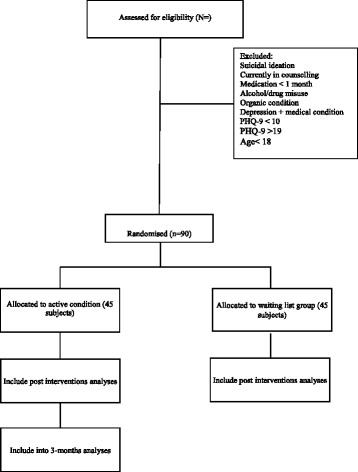
Participant flow CONSORT

After baseline screening, participants will be randomised and informed immediately about their group assignment. Randomisation will be handled by a computer algorithm administered by a person independent of the researchers. Figure 1 shows the CONSORT participant flow through the trial.

#### Intervention space from depression

*Space from Depression* programme (*Yo puedo sentirme Bien* –Spanish version) consists of seven modules of cognitive-behavioural therapy (Table 2). The treatment includes self-monitoring, behavioural activation, cognitive restructuring, and challenging core beliefs. All modules have the same structure and format, which consist of quizzes, videos, educational content, activities with homework suggestions and a module review page. Also, users have a supporter, who will give feedback asynchronously [41].

**Table 2:** Space from depression programme intervention description [41]

Module	Brief description
Getting started	This module outlines the basic premise of CBT, provides information about depression, and introduces some of the key ideas of Space from Depression. Users are encouraged to begin to chart their own current difficulties with depression.
Tune in I: getting to grips with mood	The focus in this module is on mood monitoring and emotional literacy. Users can explore different aspects of emotions, physical reactions, action and inaction, and how they are related.
Tune in II: spotting thoughts	This module focuses on noting and tracking thoughts. Users can explore the connection between their cognitions and their mood and record them graphically.
Change It I: boosting behaviour	This module focuses on behavioural change as a way to improve mood. Ideas about behavioural activation are included, and users can plan and record activities and chart their relationship with their mood.
Change It II: challenge your thoughts	This module supports users to challenge distorted or overly negative thinking patterns with thought records, as well as helpful coping thoughts.
Change It III: core beliefs	This module outlines the role that deeply held core beliefs could play in mood and depression. Users can use a range of interactive activities to identify, challenge and balance any unhelpful core beliefs.
Bringing It all together	In this final module, users are encouraged to bring together all the skills and ideas they have gathered so far, note their personal warning signs, and make a plan for staying well.

#### Wait list control group

Participants in the WL control group will receive treatment after seven weeks; therefore, this group will receive no treatment for the duration of the first seven weeks.

#### Supporters

Participants will be assigned a supporter, who will be a postgraduate student in clinical psychology with supervised experience in CBT with adults. Supporters will be trained in the *Space from Depression* (*Yo puedo sentirme bien*) platform and programme before starting their role as a supporter and will be supervised by an experienced clinical psychologist at the university. Each supporter will be assigned users and they will provide asynchronous post-session feedback of between 10 and 15 min per participant per session. The role of the supporter consists of motivating and providing feedback to the users. They can schedule the feedback at specific times once each week for a period of seven weeks.

#### Assessments

In study 1 cultural sensitivity will be determined through the ‘*top-down*’ revisions performed by the clinician researcher (AS), together with translation and customisation. Ecological validity using experts and users will assess the cultural relevance of the *Space from Depression* (*Yo puedo sentirme bien*) programme using the Cultural Relevance Questionnaire (CRQ).

In study 2, the efficacy trial, participants will be assessed at baseline, post treatment, and at 3-month follow-up. Details of the measures are described in Table 3.

**Table 3:** Study measures to be used

Study	Measure	Assessed variable	Time of assessment
Study 1 cultural validity	Cultural Relevance Questionnaire (CRQ)	Culturally adapted internet-delivered treatment	Completed by experts and users before the trial.
Study 2 efficacy trial	Patient Health Questionnaire (PHQ-9)	Depression symptoms	Baseline, post-treatment and follow- up
	Generalised Anxiety Disorder 7 (GAD-7).	Anxiety symptoms	Baseline, post-treatment and follow- up
	Sociodemographic & Clinical History Questionnaire	Gender, age, marital status, education, occupation, socioeconomic status and clinical history	Baseline
	Helpful and Hindering Aspects of Treatment (HAT)	Helpful and hindering aspects of treatment	After each session
	Satisfaction with Treatment (SAT)	Satisfaction with therapy	Post-treatment

Participants will be assessed at baseline through the Patient Health Questionnaire (PHQ-9), Sociodemographic & Clinical History Questionnaire and Generalised Anxiety Disorder-7 (GAD-7). At the beginning of each session participants will be asked to reflect on their previous session and complete the Helpful Aspects of Treatment Form (HAT). PHQ-9 and GAD-7 will be completed at week 7 and at follow-up (week 20 / 3 months). In addition, satisfaction with Treatment (SAT) will be administered at week 7.

### Measures

#### Study 1

***Cultural Relevance Questionnaire (CRQ):*** The Cultural Relevance Questionnaire is an instrument that evaluates a culturally adapted psychotherapy protocol. This questionnaire is based on cultural sensitivity and ecological validity theory by Bernal (2009) and Helms (2015) and their proposals for culturally adapted evidence-based practices. The CRQ has two sections, which includes a general assessment of the programme and an assessment of each module. Based on the Helms (2015) categories of functional equivalence, conceptual equivalence and linguistic equivalence, the first section evaluates eight areas [(a) language, (b) persons, (c) metaphors (d) content, (e) concepts, (f) goals, (g) methods and (g) context] [45] and is composed of five questions assessed on a 5-point Likert scale. The second section evaluates each modules content, examples, and personal stories, collecting qualitative observations based on the categories: functional equivalence, conceptual equivalence and linguistic equivalence.

#### Study 2

##### Screening measure

***Sociodemographic Information & Clinical History Questionnaire:*** This instrument was developed based on a previous version [55] and it collects details on the participants, such as data on the length of time that one is experiencing depression symptoms. Also, it collects data on participants’ experience of counselling/therapy and medication for depression. Data is collected on whether one has a previous diagnosis of an organic mental health disorder such as schizophrenia, psychosis and bipolar disorder. In addition, it contains items related to co-morbidity of depression with alcohol and drug misuse, and/or any recent medical diagnosis.

##### Primary outcome measure

***Patient Health Questionnaire (PHQ-9)*** [56]*:* PHQ-9 is a 9-item self-report measure that assesses the nine depression symptoms from the DSM-IV depression criteria. Each item is scored on a 4-point scale (0–3) and scores range from 0 to 27. The score can be used to describe the patient’s symptoms in one of five categories: none (0–4), mild (5–9), moderate (10–14), moderately severe (15–19), and severe (20–27). The PHQ-9 has been shown to have good reliability and validity in primary care populations, and to have good internal consistency and structural validity (α = 0.89, α = 0.86, respectively) [57]. PHQ-9 has been translated into Spanish and a Colombian version will be used in the current study (base on www.phqscreeeners.com/). The Spanish version of PHQ-9 has demonstrated reliability (α = 0.85) among Latinos in the U.S. [58, 59].

##### Secondary outcome measure

***General Anxiety Disorder (GAD-7)*** [60]*:* Comprises seven items measuring symptoms and severity of anxiety based on the DSM-IV diagnostic criteria. GAD-7 has shown reliability (α = 0.92) [61]. The GAD-7 has been culturally adapted into Spanish [62] and is available in a Spanish language Colombian version (www.phqscreeeners.com/).

##### Others measures

***Helpful Aspects of Therapy Form (HAT)*** [63, 64]*.* It is an instrument that assesses the most helpful and hindering events in the therapy. Participants are asked to describe any event or anything they engaged with in the session that was helpful or hindering for them.

***Satisfaction with Treatment (SAT)*** [65]*.* At post-treatment, participants will be asked to complete a satisfaction with treatment measure. SAT asks clients about positive and negative experiences with the internet-delivered treatment. SAT contains two questions asking participants to describe what participants most liked and least liked about the online treatment.

### Ethics

The research project and all related materials were submitted and approved by the appropriate university ethics committees in Bogota (letter reference no. 552 on December 1st 2015) and Bucaramanga (letter reference no. 075 on March 28th 2016).

### Planned analysis

#### Study 1: Cultural adaptation (cultural sensitivity and ecological validity)

Descriptive statistics will report on the results from the quantitative questions on CRQ. The CRQ data collected from the five open questions and the open questions per module will be analysed qualitatively [66]. The feedback will be categorised based on Helms [46] functional, conceptual and linguistic equivalence. The information will be assigned a colour code indicating positive and negative comments and suggestions for improving the programme. Likewise, new categories that might arise from the data will be considered. Finally, the results gathered from experts and users will be incorporated into the *Space from Depression* programme.

#### Study 2: Efficacy trial

All analyses will be based on the intention-to-treat principle. Participants’ data will be included irrespective of treatment compliance. All of the data will be prepared and reviewed in SPSS.

Descriptive statistics will be used to analyse sociodemographic variables (e.g., gender and age). Chi-squared and t-tests will be used to examine demographic differences and clinical characteristics in the groups [67].

Effects will be tested at the 0.05 level. To assess significant changes over time, repeated measures ANOVAs will be performed for the primary outcome measure for depression (PHQ-9) and secondary outcome (GAD-7) [68]. The magnitude of effects within and between the two groups will be established by Cohen’s *d* statistic [67]. This will determine what is considered a small effect (*d* ≥ 0.2), a medium effect (*d* ≥ 0.5) and a large effect (*d* ≥ 0.8) [69].

Analyses will be carried out with participants who have clinically significant changes at the end of the intervention and at follow-up. The assessments will be made using pre-treatment scores, and these will be compared to post-treatment scores and follow-up scores on the outcome measures PHQ-9 and GAD-7. The analysis will be based on the Jacobson & Truax method, where a reliable change equals the difference pre-test and post-test, divided by the standard error of the difference [70].

Linear Mixed Model will be used for repeated measures (PHQ-9, GAD7) on each participant over time; it allows for a variety of correlation patterns (variance and covariance structures) to be explicitly modelled [71]. Also, it allows the measures on each subject to be used while accounting for missing data.

Participants’ responses in the HAT, identifying helpful and non-helpful events in the therapy and their impacts will be analysed qualitatively [66]. At the beginning, individual units of text will be analysed and identified out of their context. Next, these meaning units will be organised into helpful and non-helpful events and their impacts. Finally, these will be grouped into categories, which will be suitably named and defined.

Descriptive analysis will be used to analyse quantitative data from the SAT and their qualitative responses analysed using thematic analysis.

## Discussion

This study aims to develop a robust and theoretically-informed methodology to adapt an internet-delivered intervention for depression in another culture. Research about cultural adaptation in psychotherapy is a relatively new field in psychology [46], and even more limited regarding the cultural adaptation of internet-delivered treatments [13]. The cultural relevance of the programme will be assessed through CRQ, a theoretically based measure developed for the current study, which will be completed by users and experts. The CRQ focuses on the cultural sensitivity and ecological validity of interventions protocols. This measure and methodology is one of the first research contributions about culturally adapted internet-delivered psychotherapy, which will enable knowledge development for future studies with diverse populations in other countries in South America and further field. The methodology can be further refined and elaborated upon in future studies. The inclusion of diverse populations in clinical research has been a challenge for many years. Those are generally underrepresented in scientific and clinical analyses and are known to be a hard-to-reach population for research purposes [13]. The current study supports this area and the relevant necessity to use culturally adapted internet-delivered interventions for depression.

The study further aims to evaluate the efficacy of the culturally adapted *Space from Depression* programme for college students with depressive symptoms in Colombia. The study will be a first contribution in South America regarding the potential impact of a culturally adapted internet-delivered, low-intensity intervention on depressive symptoms for college students as compared to a WL control group. Internet-delivered interventions could be a potential option for delivering evidence-based psychological intervention, and may overcome some of the significant barriers to accessing mental health treatment in Colombia.

*Space from Depression* has been investigated with college students and a community population, with significant improvements in depression pre-to-post treatment [41]. We hypothesise that we can achieve similar positive outcomes from a culturally adapted version. Most of the studies of internet-delivered interventions have been studied in HICs, which might not generalizable to other populations worldwide. Culturally adapted internet-delivered interventions are an opportunity to access and disseminate evidence-base treatments. This type of treatment as an alternative for people who cannot access mental health services due to limited health insurance, or personal stigma [72] and therefore is relevant for Colombia.

A noted limitation is that the study will not include an official depression diagnosis of participants; however, it includes well-established measures of symptom severity that can allow us to establish the efficacy of low intensity internet-delivered treatments for depression among college students in Colombia.

### Trial status

Recruitment began in August 2016. Currently, the study is in progress.

## Abbreviations

CAP: Culturally adapted psychotherapy
CBT: Cognitive behaviour therapy
CRQ: Cultural relevance questionnaire
DSM-IV: Diagnostic statistical manual, 4th edition
GAD-7: General anxiety disorder, 7-item
HAT: Helpful aspects of therapy form
HICs: High-income countries
iCBT: Internet-delivered cognitive behavioural therapy
LMCs: Low and middle income countries
PHQ-9: Patient health questionnaire, 9-item
PTSD: Posttraumatic stress disorder
RCT: Randomised controlled trial
SAT: Satisfaction with treatment
WL: Waiting-list
